# Elevated systemic inflammation response index is associated with poor outcomes in minor ischemic stroke

**DOI:** 10.3389/fneur.2024.1492224

**Published:** 2024-11-20

**Authors:** Jie Li, Ping Zhang, Hong Chen, Yanfen Wang, Yangyun Han, Chun Wang, Xingyang Yi

**Affiliations:** ^1^Department of Neurology, Deyang People’s Hospital, Deyang, China; ^2^Sichuan Clinical Research Center for Neurological Diseases, Deyang, China

**Keywords:** minor ischemic stroke, prognosis, disability, systemic inflammation response index, odds ratio

## Abstract

**Objectives:**

Patients with minor ischemic stroke (MIS) have substantial disability rates at 90 days. Our study aimed to explore the association between the systemic inflammation response index (SIRI) and 3-month functional outcomes in patients with MIS.

**Methods:**

We conducted a prospective observational study in patients with MIS [defined as a National Institutes of Health Stroke Scale (NIHSS) score of 0–3] admitted within 24 h from symptoms onset. Blood samples for the SIRI measurement were collected on admission. The primary outcome measure was poor outcomes at 90 days (defined as a modified Rankin Scale score of 2–6). Univariate and multivariate logistic analyses were performed to assess the association between the SIRI and the risk of 3-month poor outcomes.

**Results:**

A total of 152 patients with MIS were enrolled, of which 24 cases (15.8%) had poor outcomes at 90 days. The median SIRI level was 1.27 [interquartile range (IQR), 0.77–1.92, ×10^9 /L] on admission. MIS patients with poor outcomes had higher levels of the SIRI than patients with good outcomes (poor outcomes: median, 1.93, IQR: 1.17–3.28, ×10^9 /L; good outcomes: median, 1.21, IQR: 0.71–1.80, ×10^9 /L; *p* = 0.003). The high SIRI level group (SIRI >1.27 × 10^9 /L) had significantly higher rates of poor outcomes at 90 days (22.4% vs. 9.2%, *p* = 0.026). After adjusting for age, baseline NIHSS score, prehospital delay, Trial of Org 10,172 in Acute Stroke Treatment (TOAST) classification, and other confounders in multivariate analyses, an elevated SIRI level remained independently associated with an increased risk of poor outcomes in patients with MIS [odds ratio (OR): 1.57, 95% confidence interval (CI): 1.12–2.20; *p* = 0.010]. Meanwhile, a high level of the SIRI (>1.27 × 10^9/L) was still an independent risk factor for 3-month poor outcomes (OR: 4.80, 95%CI: 1.51–15.29; *p* = 0.008) in MIS patients.

**Conclusion:**

Disability at 90 days was common in patients with MIS. An elevated SIRI was associated with poor outcomes in MIS patients. The SIRI might be a promising biomarker candidate that can help identify high-risk MIS patients with poor outcomes for reaching individual therapeutic decisions in clinical trials.

## Introduction

Minor ischemic stroke (MIS) is fairly common and accounts for approximately 30% of all strokes (1). Although the outcomes of most patients with MIS are favorable, there are still a few individuals who suffer poor outcomes (2). Several studies suggested that approximately 11–34% of patients with MIS had significant disability at hospital discharge (3–6). It also has been demonstrated that patients with MIS have substantial rates (29%) of disability at 90 days (7). Therefore, it is essential to identify the MIS patients who are at a higher risk of poor outcomes in the early stage of clinical practice.

It has been demonstrated that brain inflammation might continuously shape pathophysiological processes after cerebral ischemic injury and is closely linked to the development, progression, and outcome of acute ischemic stroke (AIS). Inflammation can induce secondary brain injury by exacerbating blood–brain barrier damage, leukocyte infiltration, secretion of multiple inflammatory mediators, microvascular failure, brain edema, and neuronal cell death (8–11). Therefore, inflammation is currently considered to be one of the major targets for developing new stroke therapies, with broad application prospects (12, 13). According to a recent systematic review, blood-based biomarkers of inflammation are expected to be one of the most promising biomarkers to predict functional outcomes in stroke patients (14). As inflammatory indicators composed of the ratios of blood cell subgroups, neutrophil-to-lymphocyte ratio, platelet-to-lymphocyte ratio, and lymphocyte-to-monocyte ratio have been extensively studied as prognostic tools (15–18); however, the results of some studies were inconsistent (17, 18). The systemic inflammation response index (SIRI) is a new and more comprehensive marker based on the composition ratio of peripheral blood neutrophil, monocyte, and lymphocyte counts (calculated by neutrophil count × monocyte count/lymphocyte count) (19). Several studies have investigated the relationship between the SIRI and the outcomes of patients with AIS (19–25). A retrospective study based on the Medical Information Mart for Intensive Care IV (MIMIC-IV) database found that an elevated SIRI was associated with a higher risk of mortality among intensive care unit patients with AIS (19). Some studies have suggested that higher admission SIRI was associated with poor functional outcomes of AIS (20–24), especially for those AIS patients who were treated with intravenous thrombolysis or endovascular therapy (20–23), but another study has reached a different conclusion (25). Fewer studies have attempted to investigate the association between the SIRI and the prognosis in patients with MIS.

To date, whether SIRI levels are associated with functional outcomes in patients with MIS has not been elucidated. Therefore, the aim of the current study was to explore the potential association between SIRI levels and functional outcomes in patients with MIS.

## Methods

### Study design and subjects

A total of 152 consecutive MIS patients who had magnetic resonance diffusion-weighted imaging (DWI) evidence of new-onset cerebral infarction and were hospitalized within 24 h from symptoms onset were enrolled in this observational study from 1 March 2020 to 31 June 2021. MIS was defined as a baseline total National Institutes of Health Stroke Scale (NIHSS) score of ≤3 (26). All patients received an extensive stroke etiologic workup and were routinely followed up via telephone or mail after 3 months. We excluded cases with incomplete hospital records or missing imaging data. We also excluded cases with a pre-stroke modified Rankin Scale (mRS) score of ≥2 and those who lived with disabilities (27). Meanwhile, patients who had premorbid conditions such as infections, connective tissue diseases, malignancies, or other disorders that might affect the blood system were excluded as well. Detailed methods for including and excluding patients and the flow diagram have been described in our previous study (28). The study protocol was approved by the Ethics Committee of Deyang People’s Hospital (Reference No. 2019–01-142-K01) and registered (unique Identifier: ChiCTR2000029902).^1^ Written informed consent was obtained from all patients before they were enrolled.

### Data collection

We collected patient data using a standardized form that included age, sex, prehospital delay, baseline NIHSS score, systolic and diastolic blood pressure on admission, baseline serum glucose, vascular risk factors, and potential stroke etiology, which have been elaborated in our previous study (29). In-hospital treatments analyzed in our study included intravenous thrombolysis, antiplatelet therapy, antihypertensives, antidiabetics, and statins. Intravenous thrombolysis was performed according to the Chinese guidelines, which had similar inclusion and exclusion criteria compared to the American guidelines (30, 31). The final treatment decision was made in consultation with a neurologist and the patient’s family. Antiplatelet therapies were administered at the physicians’ discretion. Patients included in the present study received either (1) aspirin or clopidogrel only, or (2) clopidogrel plus aspirin (dual antiplatelet therapy) at admission.

### Laboratory measurements and SIRI levels

Blood samples were collected on admission from the cubital vein from each patient before initial treatment. The absolute counts of white blood cell subgroups such as lymphocytes, monocytes, and neutrophils were assessed, as well as C-reactive protein (CRP). We calculated the level of SIRI as follows: SIRI = neutrophil count × monocyte count/lymphocyte count (19).

### Assessment of clinical outcomes

The degree of disability was measured by using a modified Rankin Scale (mRS) score at 90 days after admission (27). The primary outcome of the current study was poor outcomes at 90 days, defined as a mRS score of 2–6 (7). The secondary outcome was 3-month mortality and recurrent ischemic stroke during the first 3 months after admission.

### Statistical analyses

Continuous variables are presented as means with standard deviations (SD) or medians with interquartile ranges (IQR), and categorical variables are presented as frequencies with percentages. The normality of data was tested using a Shapiro–Wilk test. The *χ*^2^ tests or Fisher’s exact tests were used for differences in categorical data, while Student’s *t*-tests or the Mann–Whitney *U*-test were used for differences in continuous data. Baseline characteristics, laboratory values, and in-hospital treatment were compared between MIS patients with good or poor outcomes.

Univariate analyses comparing the baseline characteristics and clinical outcomes between low SIRI and high SIRI level groups were performed. Multivariate logistic regression analyses were performed to identify the association between the SIRI and 3-month poor outcomes of MIS patients in three different models. Model 1 was adjusted for age, baseline NIHSS score, and prehospital delay using the forward logistic regression (LR) method. Model 2 was adjusted for variables in model 1 and variables that had a potential association with 3-month poor outcomes in univariate analyses (*p* < 0.05). Model 3 was adjusted for variables in model 1, TOAST classification, and variables that had a potential association with 3-month poor outcomes in univariate analyses (*p* < 0.05).

All statistical analyses were performed using SPSS v21.0 (IBM, Chicago, IL, United States), the statistical software packages R (The R Foundation, version 3.4.3),^2^ and EmpowerStats (X&Y Solutions, Inc., Boston, MA, United States),^3^ which have been described in our previous study (32). A two-sided *p*-value of <0.05 was considered to be statistically significant.

## Results

During the study period, 798 AIS patients without a pre-stroke mRS score of ≥2 were consecutively registered. Of these, 152 (19.0%) who were admitted within 24 h were enrolled in the study [mean age: 67.7 ± 11.1 years; 104 (68.4%) male; median baseline NIHSS score: 2, IQR, 1–3]. The median SIRI level was 1.27 [interquartile range (IQR), 0.77–1.92] on admission. Overall, 30 (19.7%) cases were treated with intravenous thrombolysis, and 109 (71.7%) cases were treated with dual antiplatelet therapy after admission. All enrolled patients completed a 3-month follow-up. Three (2.0%) patients died, and 24 cases (15.8%) had poor outcomes (mRS score of 2–6) at 90 days.

### Baseline characteristics and in-hospital treatment between MIS patients with good or poor outcomes

**Figure 1:**
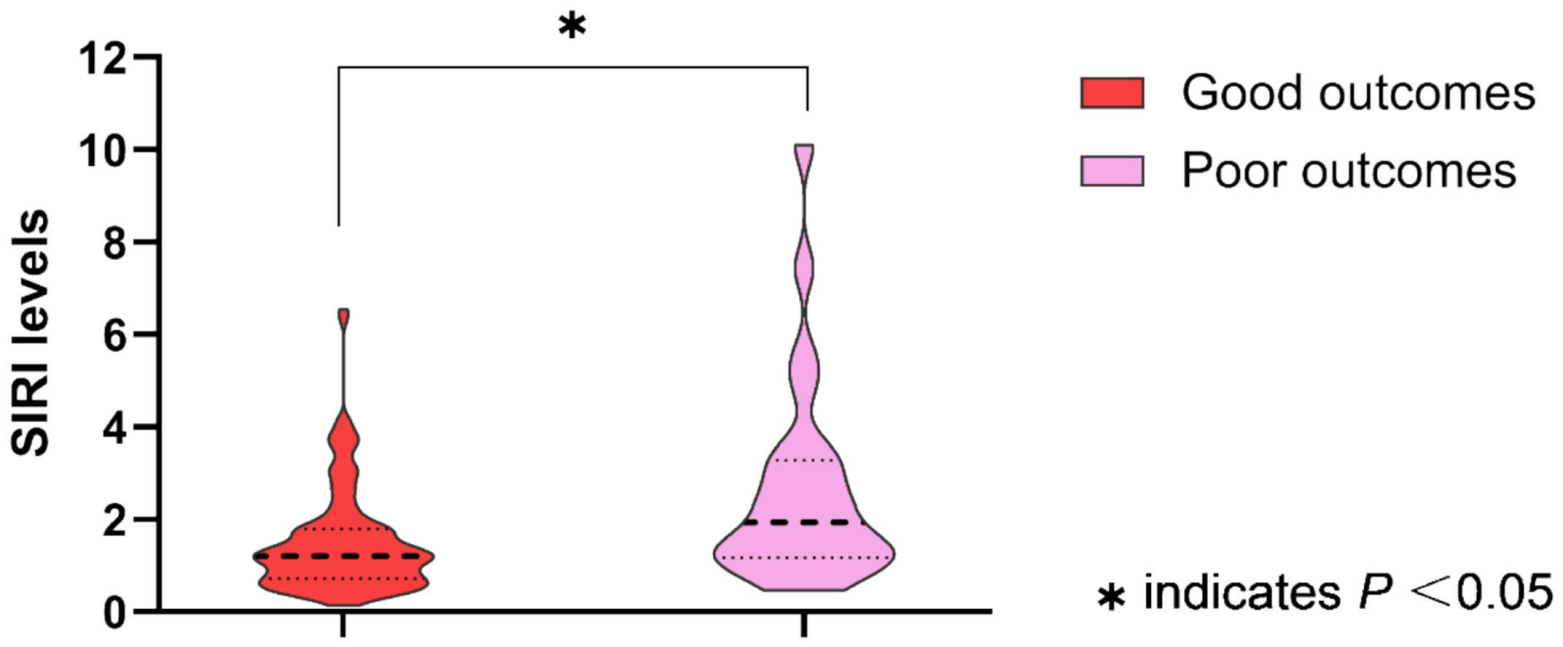
Systemic inflammation response index (SIRI) levels between groups are shown as violin plots (MIS patients with good outcomes vs. poor outcomes, *p* = 0.003).

The baseline characteristics, in-hospital treatment, and the median SIRI levels in MIS patients with good or poor outcomes are summarized in Table 1. MIS patients with poor outcomes had higher levels of the SIRI than patients with good outcomes (poor outcomes: median, 1.93, IQR, 1.17–3.28; good outcomes: median, 1.21, IQR, 0.71–1.80; *p* = 0.003). The SIRI levels between groups are shown as violin plots in Figure 1. Meanwhile, as compared to the good outcome group, patients with poor outcomes were older (73.4 ± 11.1 vs. 66.6 ± 10.9, *p* = 0.006) and less frequently received dual antiplatelet therapy (50.0% vs. 75.8%, *p* = 0.010). There was no difference in the sex, prehospital delay, the NIHSS score on admission, baseline systolic and diastolic blood pressure, baseline serum glucose, vascular risk factors, TOAST classification, other laboratory values, and other in-hospital treatments between the two groups (all *p* > 0.05).

**Table 1:** Baseline characteristics and in-hospital treatment between MIS patients with good or poor outcomes.

	Good outcomes (*N* = 128)	Poor outcomes (*N* = 24)	*p-*value
Age, years	66.6 ± 10.9	73.4 ± 11.1	0.006*
Sex (male)	85 (66.4)	19 (79.2)	0.217‡
Prehospital delay, hours	8.5 (3.0–22.0)	7.5 (3.0–10.8)	0.405†
NIHSS score on admission	2 (1–3)	2 (2–3)	0.605†
SBP on admission (mm Hg)	158.2 ± 25.6	156.6 ± 24.9	0.785*
DBP on admission (mm Hg)	89.3 ± 14.2	85.4 ± 15.3	0.222*
Baseline serum glucose (mmol/L)	8.9 ± 4.7	9.6 ± 4.2	0.513*
Risk factors			
Hypertension	106 (82.8)	19 (79.2)	0.890‡
Diabetes mellitus	44 (34.4)	10 (41.7)	0.493‡
Dyslipidemia	46 (35.9)	6 (25.0)	0.300‡
Coronary heart disease	13 (10.2)	3 (12.5)	1.000‡
Atrial fibrillation	23 (18.0)	2 (8.3)	0.385‡
Rheumatic heart disease	4 (3.1)	0 (0)	1.000#
Current smoking	68 (53.1)	15 (62.5)	0.397‡
Previous ischemic stroke	19 (14.8)	3 (12.5)	1.000‡
Previous ICH	6 (4.7)	0 (0)	0.590‡
TOAST classification			0.211‡
Large-artery atherosclerosis	38 (29.7)	11 (45.8)	
Cardioembolism	16 (12.5)	0 (0)	
Small-vessel occlusion	58 (45.3)	12 (50.0)	
Other determined etiology	2 (1.6)	0 (0)	
Undetermined etiology	14 (10.9)	1 (4.2)	
Laboratory values, median (IQR)			
Neutrophils, ×10^9 /L	4.12 (3.29–5.53)	5.16 (3.54–6.95)	0.077†
Lymphocytes, ×10^9 /L	1.53 (1.16–1.95)	1.12 (0.89–1.96)	0.053†
Monocytes, ×10^9 /L	0.40 (0.31–0.52)	0.45 (0.36–0.68)	0.114†
CRP (mg/L)	0 (0–1.76)	1.03 (0–20.82)	0.266†
SIRI, ×10^9 /L	1.21 (0.71–1.80)	1.93 (1.17–3.28)	**0.003**†
In-hospital treatment			
Intravenous thrombolysis	22 (17.2)	8 (33.3)	0.123‡
Dual antiplatelet therapy	97 (75.8)	12 (50.0)	**0.010‡**
Antihypertensives	70 (54.7)	8 (33.3)	0.055
Antidiabetics	36 (28.1)	8 (33.3)	0.606
Statins	128 (100)	24 (100)	–

Data are represented as number (%), mean ± SD, or median and IQR. SD, standardized difference; IQR, interquartile range; NIHSS, National Institutes of Health Stroke Scale; SBP, systolic blood pressure; DBP, diastolic blood pressure; TOAST, Trial of Org 10,172 in Acute Stroke Treatment; ICH, intracerebral hemorrhage; CRP, C-Reactive Protein; SIRI, systemic inflammation response index. *Student t-test. †Mann–Whitney U-test. ‡χ^2^ test. #Fisher’s exact test. The bold values indicates *p* < 0.05.

### Baseline characteristics and clinical outcomes between low SIRI and high SIRI level groups

**Figure 2:**
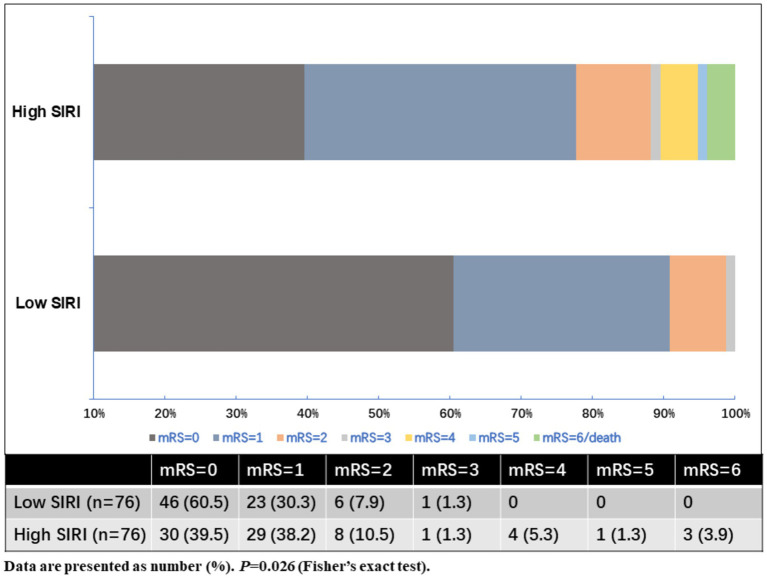
Distributions of the mRS score at 3 months for MIS patients between low SIRI and high SIRI level groups.

As shown in Table 2, MIS patients were divided into two groups according to the baseline SIRI levels. The high SIRI level group was defined as cases who had baseline SIRI levels greater than the median SIRI levels (>1.27), while the low SIRI level group was defined as cases who had baseline SIRI levels less than or equal to the median SIRI levels (≤1.27). Compared to the low SIRI level group, patients in the high SIRI level group had longer prehospital delay (11.5 vs. 6.0 h, *p* < 0.001). As for the laboratory data, the high SIRI level group had higher levels of neutrophil counts and monocyte counts, and lower levels of lymphocyte counts (all *p*-values <0.001). There was no difference in age, sex, initial NIHSS score on admission, baseline systolic and diastolic blood pressure, baseline serum glucose, vascular risk factors, TOAST classification, CRP levels, or in-hospital treatment between the high and low SIRI level groups (all *p* > 0.05). For the clinical outcomes, the high SIRI level group (SIRI >1.27) had a significantly higher rate of poor outcomes at 90 days (22.4% vs. 9.2%, *p* = 0.026), and there was no difference in the incidence rate of recurrent ischemic stroke or 3-month mortality between the two groups (all *p* > 0.05). The distributions of the mRS score at 3 months for MIS patients between low SIRI and high SIRI level groups are displayed in Figure 2.

**Table 2:** Baseline characteristics and clinical outcomes between low SIRI and high SIRI level groups.

	Low SIRI (≤1.27) (*n* = 76)	High SIRI (>1.27) (*n* = 76)	*p-*value
Age, years	66.2 ± 10.7	69.1 ± 11.5	0.111*
Sex (male)	48 (63.2)	56 (73.7)	0.163 ‡
Prehospital delay, hours	6.0 (2.3–10.0)	11.5 (5.0–24.0)	**<0.001†**
NIHSS score on admission	2 (1–3)	2 (1–3)	0.528†
SBP on admission (mm Hg)	159.6 ± 26.5	156.2 ± 24.4	0.413*
DBP on admission (mm Hg)	89.7 ± 13.8	87.7 ± 15.0	0.384*
Baseline serum glucose (mmol/L)	9.3 ± 4.8	8.7 ± 4.5	0.448*
Risk factors			
Hypertension	58 (76.3)	67 (88.2)	0.056‡
Diabetes mellitus	29 (38.2)	25 (32.9)	0.498‡
Dyslipidemia	28 (36.8)	24 (31.6)	0.494‡
Coronary heart disease	6 (7.9)	10 (13.2)	0.290‡
Atrial fibrillation	10 (13.2)	15 (19.7)	0.274‡
Rheumatic heart disease	2 (2.6)	2 (2.6)	1.000‡
Current smoking	40 (52.6)	43 (56.6)	0.625‡
Previous ischemic stroke	10 (13.2)	12 (15.8)	0.645‡
Previous ICH	3 (3.9)	3 (3.9)	1.000‡
TOAST classification			0.051#
Large-artery atherosclerosis	24 (31.6)	25 (32.9)	
Cardioembolism	7 (9.2)	9 (11.8)	
Small-vessel occlusion	40 (52.6)	30 (39.5)	
Other determined etiology	2 (2.6)	0 (0)	
Undetermined etiology	3 (3.9)	12 (15.8)	
Laboratory values, median (IQR)			
Neutrophils, ×10^9 /L	3.66 (2.94–4.30)	5.27 (4.12–7.40)	**<0.001†**
Lymphocytes, ×10^9 /L	1.68 (1.24–2.17)	1.23 (0.99–1.69)	**<0.001†**
Monocytes, ×10^9 /L	0.35 (0.29–0.42)	0.51 (0.40–0.67)	**<0.001†**
CRP (mg/L)	0.53 (0–2.51)	0 (0–1.24)	0.309†
In-hospital treatment			
Intravenous thrombolysis	19 (25.0)	11 (14.5)	0.103†
Dual antiplatelet therapy	58 (76.3)	51 (67.1)	0.207†
Antihypertensives	38 (50.0)	40 (52.6)	0.746†
Antidiabetics	25 (32.9)	19 (25.0)	0.283†
Statins	76 (100)	76 (100)	–
Recurrent ischemic stroke	2 (2.6)	1 (1.3)	1.000†
3-month mortality	0 (0)	3 (3.9)	0.244†
3-month poor outcome	7 (9.2)	17 (22.4)	**0.026**‡

Data are number (%), mean ± SD, or median and IQR. SD, standardized difference; IQR, interquartile range; NIHSS, National Institutes of Health Stroke Scale; SBP, systolic blood pressure; DBP, diastolic blood pressure; TOAST, Trial of Org 10,172 in Acute Stroke Treatment; ICH, intracerebral hemorrhage; CRP, C-Reactive Protein; SIRI, Systemic Inflammation Response Index. *Student t-test. †Mann–Whitney U-test. ‡χ^2^ test. #Fisher’s exact test. The bold values indicates *p* < 0.05.

### Multivariate analyses for the association between SIRI and 3-month poor outcomes in MIS patients

Multivariate logistic regression analyses were performed to identify the association between SIRI and 3-month poor outcomes of MIS patients in three different models, as shown in Table 3. After adjusting for age, baseline NIHSS score, and prehospital delay (model 1), both elevated SIRI level (*p* = 0.005) and high SIRI level groups (*p* = 0.025) were significantly associated with 3-month poor outcomes in MIS patients. When variables that had a potential association with 3-month poor outcomes in univariate analyses were further added in the multivariate logistic regression (model 2), both elevated SIRI level (*p* = 0.005) and high SIRI level groups (*p* = 0.039) were also significantly associated with 3-month poor outcomes. After adjusting for age, baseline NIHSS score, prehospital delay, TOAST classification, and other potential confounders (model 3), elevated SIRI level remained independently associated with an increased risk of poor outcomes [odds ratio (OR): 1.57, 95% confidence interval (CI): 1.12–2.20; *p* = 0.010]. Meanwhile, a high level (>1.27) of SIRI was still an independent risk factor for 3-month poor outcomes (OR: 4.80, 95%CI: 1.51–15.29; *p* = 0.008).

**Table 3:** Multivariate analysis between SIRI and 3-month poor outcomes in MIS patients.

Variables	SIRI levels		High SIRI group	
	OR (95%CI)	*p-*value	OR (95%CI)	*p-*value
Unadjusted	1.57 (1.18–2.08)	**0.002**	2.84 (1.10–7.32)	**0.031**
Model 1	1.56 (1.15–2.12)	**0.005**	3.20 (1.16–8.81)	**0.025**
Model 2	1.54 (1.14–2.08)	**0.005**	3.00 (1.06–8.51)	**0.039**
Model 3	1.57 (1.12–2.20)	**0.010**	4.80 (1.51–15.29)	**0.008**

Model 1: adjusted for age, baseline NIHSS score, and prehospital delay using the forward LR method. Model 2: adjusted for age, baseline NIHSS score, prehospital delay, and variables that had a potential association with 3-month poor outcomes in univariate analyses (*p* < 0.05) using the forward LR method. Model 3: adjusted for age, baseline NIHSS score, prehospital delay, TOAST classification, and variables that had a potential association with 3-month poor outcomes in univariate analyses (*p* < 0.05) using the forward LR method. OR, odds ratios; CI, confidence intervals; SIRI, Systemic Inflammation Response Index. The bold values indicates *p* < 0.05.

## Discussion

In China, there are approximately 3 million new-onset cases of stroke each year, with approximately 1 million of these cases being MIS (33, 34). Since the initial stroke severity assessed by the NIHSS score is the most crucial prognostic indicator for stroke patients, the prognosis of MIS patients is generally good (2). However, prospective observational studies have shown that 4.5 to 26.4% of MIS patients experience early neurological deterioration and develop disability (7, 35–37). In addition, approximately 11 to 34% of MIS patients had significant disability at the time of discharge (3–6). It also has been demonstrated that the incidence rate of disability in MIS patients can be as high as 29% at 90 days (7, 38, 39). Our study provides further evidence that disability at 90 days was common in patients with MIS. The incidence rate of disability at 90 days in patients with MIS is 15.8% in our cohort. The differences in the incidence of poor outcomes in patients with MIS may reflect the heterogeneity in the demographics of enrolled patients (age, sex, and race), prehospital delay, the definition of MIS, and the way in which poor outcomes of MIS patients are defined, highlighting the need for a standardized definition of MIS. Regarding the risk factors associated with unfavorable outcomes in MIS patients, several factors have been proposed, including advanced age, female sex, increased baseline NIHSS score, stroke etiology, early neurological deterioration, acute infarct growth, the penumbra volume (>5 cm^3^) on computed tomography perfusion, and medication adherence (7, 38–42). However, to date, the risk factors associated with poor outcomes in patients with MIS have not been clearly elucidated. A proper understanding of the factors associated with unfavorable outcomes in patients with MIS could provide valuable insights for close monitoring of MIS patients. Moreover, early targeting of patients at a higher risk of poor outcomes is of great importance for improving the outcome of MIS.

In the present study, we found that MIS patients with poor outcomes had a higher level of SIRI than patients with good outcomes (1.93 vs. 1.21, ×10^9 /L). The high SIRI level group (defined as cases who had a baseline SIRI level greater than the median SIRI level, >1.27 × 10^9/L) had a significantly higher rate of poor outcomes at 90 days (22.4% vs. 9.2%). The SIRI is a new and more comprehensive inflammatory marker based on the composition ratio of peripheral blood neutrophil, monocyte, and lymphocyte counts (19). In our study, the high SIRI level group had higher levels of neutrophil counts and monocyte counts and lower levels of lymphocyte counts. After the onset of ischemic stroke, the neutrophils are the first innate immune cells infiltrating into the brain lesions through adhesion to the endothelial cells and migration (43). Neutrophils can aggravate the inflammation of the brain by releasing a variety of pro-inflammatory mediators, leading to secondary injury of brain tissue (44). Cerebral ischemia and hypoxia injury can stimulate monocytes to generate interleukin-6 (IL-6), tumor necrosis factor (TNF), and other inflammatory mediators, which further aggravate cerebral ischemia and hypoxia. Monocytes can also activate platelets to become platelet–monocyte aggregates and promote thrombosis and cerebral vascular occlusion, causing hemodynamic changes and exacerbating cerebral ischemia injury (45). Lymphocytes are involved in coordinating the inflammatory response. The role of lymphocytes in stroke is complicated. T regulatory cells (Tregs) are usually involved in inhibiting inflammation and regulating and maintaining peripheral immune tolerance and homeostasis. Moreover, Tregs secreting cytokine IL-10 have a protective effect on stroke. Experiments have demonstrated that animals with increased numbers of Tregs after stroke show better outcomes (46). The results of our study suggested that inflammation in the early stage of stroke might play an important role in the development of poor functional outcomes in MIS. Our findings also support the view that inflammation is one of the major targets for developing new stroke treatments in MIS patients in the future. As we know, a recently published randomized controlled trial (Colchicine in High-risk Patients with Acute Minor-to-moderate Ischemic Stroke or Transient Ischemic Attack, CHANCE-3) showed that the anti-inflammatory agent colchicine could not reduce the risk of subsequent stroke and poor functional outcomes (mRS > 1) within 90 days among patients with acute non-cardioembolic minor-to-moderate ischemic stroke or transient ischemic attack (47). The CHANCE-3 trial included patients with a baseline concentration for high-sensitivity C-reactive protein at least 2 mg/L (47). The negative results of the CHANCE-3 trial suggested that the optimal anti-inflammatory strategy in MIS and the biomarkers most suitable for screening high-risk MIS patients with poor outcomes still need to be further explored.

A recently published systematic review suggests that blood-based inflammatory markers might be among the most promising biomarkers for predicting functional outcomes in stroke patients (14). The SIRI, which is a more comprehensive inflammatory indicator based on the composition ratio of blood cell subgroups, has lately been explored as a novel prognostic marker for stroke (19–25, 48–49). Several studies have suggested an association between baseline SIRI level and functional outcome in patients with AIS (20–24, 49), especially for those cases treated with intravenous thrombolysis or endovascular therapy (20–23, 49), but another study came to a different conclusion (25). As a comprehensive, easily accessible, and inexpensive inflammatory marker, the SIRI might be a suitable candidate biomarker of clinical outcomes in MIS patients. However, fewer studies have attempted to elucidate the association between SIRI and outcomes in MIS. In our study, after adjusting for age, baseline NIHSS score, prehospital delay, TOAST classification, and other potential confounders in multivariate logistic regression analyses, elevated SIRI levels remained independently associated with an increased risk of poor outcomes in patients with MIS (OR: 1.57, 95% CI: 1.12–2.20). Meanwhile, a high level of the SIRI (>1.27 × 10^9/L) was still an independent risk factor for 3-month poor outcomes (OR: 4.80, 95%CI: 1.51–15.29). Our study suggested that elevated SIRI levels were associated with poor outcomes in Chinese patients with MIS. As a result, the SIRI might be a promising biomarker candidate that can help identify high-risk MIS patients with poor outcomes for reaching individual therapeutic decisions in clinical trials. Further studies with large sample sizes are needed to determine the optimal cutoff value of the SIRI as an indicator for poor outcomes in MIS and validate the SIRI as a biomarker for disability in patients with MIS.

### Limitations

There are still several limitations in the present study. Thus, the results of our study should be interpreted with caution. First, it was a single hospital-based study conducted in China; the results of our study might not be generalizable to diverse populations with different genetic, demographic, and socioeconomic backgrounds. Second, the sample size of our study was small, and finally, only 24 cases suffered poor outcomes at 90 days. Although the findings are statistically significant, the small sample size may limit the generalizability and robustness of the results. Further multicenter studies with larger sample sizes are needed to confirm these findings and determine the best cutoff value of the SIRI as a predictor of poor outcomes in patients with MIS. Third, the SIRI levels may change dynamically after acute ischemic stroke. In the current study, the SIRI level was tested only one time at baseline. We did not have longitudinal data on SIRI levels during their 90-day follow-up. Further studies are needed to evaluate the association between the dynamic changes in SIRI levels and poor outcomes in MIS patients. In addition, several studies have shown an association between inflammatory biomarkers, such as high-sensitivity C-reactive protein, cytokines, and other acute-phase proteins, and poor outcomes in AIS. However, these biomarkers were not measured in the present study. In addition, our study focused only on the 90-day outcomes, but long-term follow-up would help to understand the full impact of the SIRI on disability and survival in patients with MIS. Moreover, we performed the follow-up by telephone interviews or mailed questionnaires instead of clinic visits, which might result in reporting bias. Finally, our study was only an observational study; no causal link could be drawn. Thus, well-designed multicenter studies with large sample sizes are needed to validate our findings.

## Conclusion

Despite the above limitations, we conducted a prospective observational study in MIS patients hospitalized within 24 h from symptoms onset and identified that disability at 90 days was common in patients with MIS. An elevated SIRI was associated with poor outcomes in MIS patients. SIRI might be a promising biomarker candidate that can help identify high-risk MIS patients with poor outcomes for reaching individual therapeutic decisions in clinical trials.

## Data Availability

The raw data supporting the conclusions of this article will be made available by the authors, without undue reservation.

## Footnotes

^1^ https://www.chictr.org.cn/

^2^ http://www.R-project.org

^3^ http://www.empowerstats.com
